# Dr. James Bradley, on Strangulated Hernia

**Published:** 1810-09

**Authors:** 


### Dr. James Bradley, on Strangulated Hernia.

( Concluded from our last, pp. 112—121. )

THERE are two prominent varieties in the phenomena of strangulated hernia, which are peculiarly deserving of attention, and which, perhaps, are dependent on the degree of stricture in the parts strangulated, as well as on some peculiarity in the constitution. The first is, where the violence of the stricture is such, combined probably with other causes, as to occasion excessive and continued sickness, which fails not to arrest the action of the heart and arteries. The circulation is of course lessened, and the patient becomes cold, ghastly, and dejected. He sighs, he moans, the tunica conjunctiva and countenance are extremely pallid, the eyes sunk within their sockets, the nostrils contracted, and the living principle is nearly extinct, before the surgeon is often aware of the danger. Here the pulse are at first slow, soft, and sometimes full; but as the disorder advances, they become more contracted and generally quicker; and if the stricture be removed before the powers of nature are too much exhausted, they will be increased for a while, both in force and velocity; but if on reduction, they fail to rise, and only increase in quickness, then considerable danger is to be apprehended.

Under every shade of this variety, the operation of opening the sac is attended with considerable risk. Under every aspect of the second variety, there is more force and action in the pulse, more general heat, freer intervals from sickness, and fewer symptoms of asthenia. The pulse, under these circumstances, are quicker, and generally more tense, and in the middle stage of the complaint, are from 100 to 120; and if the stricture be overcome, without a division of the sac, they will decrease suddenly, both in number and in force; and the operation of opening the sac, in the usual manner, if not delayed too long, will in general succeed. Here the surgeon can oftener form, though still an incorrect, yet rather a better idea as to the state of the parts concerned in the hernia, than in a case of the first variety, where the greatest mischief sometimes exists, although not clearly indicated by any external criteria. It is in cases of this kind, (meaning the first class of symptoms) that delays are of the greatest moment; and it is also under circumstances like these, that the debilitating plan should be guarded against. Bleeding is mostly injurious, and purgatives can have no other effect than increasing the sickness; and the use of cold applications are less indicated, on account of their sedative effects, and the general coldness they cannot fail to increase. The propriety of the tobacco-clyster is doubtful, from its sedative powers; especially, where the pulse are very slow and languid, and the sickness extreme; for this state is very similar to that produced by the action of tobacco itself. In most of the cases, where I have seen the tobacco-clyster administered, and where it failed, (which it generally does) it did harm, either by increasing inflammation or debility.

If the above classification of the symptoms peculiar to strangulated hernia be founded on the broad basis of observation, it is obvious, that the difference of opinion as to some of the means usually employed in its reduction is easily reconcileable. The propriety of bleeding has long divided the opinion of practitioners, and is a subject of dispute not yet fully settled, and which is, perhaps, owing more to the want of a proper arrangement, or discrimination of the phenomena attendant on this affection, than to any other cause.

Although bleeding in the first class of symptoms be generally inadmissible, yet sometimes cases occur, where it may not be improper. For instance, when the pulse is full, though the vomiting and sickness be considerable, drawing off a moderate quantity of blood, will seldom do harm, either by increasing sickness, or debility; but under most other circumstances of this variety of the complaint, I believe it will be inadviseable.

In the second class of symptoms, blood-letting may oftener be had recourse to, but even here, it ought to be employed with considerable caution; for where the parts have suffered severely from strangulation, or are in a state of inflammation, bordering on gangrene, and where the powers of life are weakened, which is known by a low, feeble, and sometimes a quick contracted pulse, together with other symptoms of debility, this remedy had better be omitted.

On the subject of blood-letting, three distinct objects present themselves to the mind of the surgeon. The first is, the probability of removing the stricture, or relaxing the tendon. The second is, to abate local inflammation within the hernial sac; and the third is, the influence which this remedy may have over the health of the patient, subsequent to the operation for bubonocele.

Although bleeding in the first intention be seldom successful, yet, if productive of no immediate or remote injury, jury, it may always be adviseable to employ it; but if on the other hand, we be certain, that under some circumstances it is capable of doing harm, it will always be necessary to exercise some caution and judgment, before its adoption.

As to the second intention, or the influence which bleeding may have in abating local inflammation, we must observe, that as long as the stricture continues, the mischief will proportionally, and progressively increase; therefore, this operation can have little or no effect, as long as the cause continues to prevail.

As to the operation for the strangulated hernia being rendered more precarious, in consequence of previous phlebotomy, it is a subject of vast importance, and on which hinges, in a great measure, the propriety of bleeding at all; but on this subject, surgeons of the present day are not perfectly agreed. Mr. Wilmer says, that bleeding renders the operation more dangerous. This theory, though not universally, yet I believe is in general true, when the practice is carried to the extent which is usually recommended.

It is much to be wished, that some surgeon who hath great opportunities for observation, would devote his attention particularly to this subject, and point out the circumstances, under which phlebotomy is, or is not, improper in this affection; as this would always be a very important directory, especially for young surgeons, who are guided more by authority than their own judgment. Mr. Hey is quite undecided on this subject, and in his comment on Mr. Wilmer's assertion, that bleeding increases the danger of the operation, he advances as a reason to the contrary, the circumstance of the patient dying under symptoms of ileus, or an inflammatory affection of the intestines; and that these could not have been caused from bleeding. That symptoms of inflammation do appear, on dissection, in the intestines after death, as Mr. Hey hath represented, is not denied; and that these appearances are occasioned by previous phlebotomy is an absurdity will be universally admitted, but that they point out on the other hand, the necessity of blood-letting, previous to the operation of bubonocele, is extremely questionable, except it can be demonstrated, that these symptoms are purely the effect of stricture; and even here, there would be many exceptions arising out of the circumstances of the case; but we know, as has before been stated, that either a new set, or an aggravation of the old symptoms, succeed the division of the sac, and we also have reasons to apprehend, that the use of the tobacco-clyster hath some share in giving rise to symptoms, which, especially after the operation of opening the sac, will be more fully displayed; and besides, it is well known, that death will bring about considerable changes in the aspect of morbid appearances. Parts that were slightly inflamed in the living, will exhibit all the signs of high inflammation, or even extravasation, in the dead subject. Another circumstance worthy of remark, is, that ileus or inflammation of the intestines is generally superinduced on the opening of the sac, and it rarely happens, on the other hand, that this affection alone proves fatal, after the stricture is removed, without a division of the sac. It therefore follows, that these inflammatory appearances afford no infallible data for determining on phlebotomy, previous to the operation of bubonocele. For these, and other reasons, already advanced, Mr. Hey's objection does not, in my opinion, satisfactorily or conclusively bear on Mr. Wilmer's position, which is certainly much too unqualified.

Purgatives, I still think, may be adviseable under some circumstances of this complaint, particularly in the second class of symptoms above specified; and especially when the vomiting is not frequent, and the intervals tolerably free from sickness. Such as are recommended in my last communication, may not be improper; if, however, they should either increase the sickness or vomiting, they ought no longer to be persisted in.

As to opium, in the cases where I have employed it, I could not discover that it any way contributed towards removing the stricture; yet notwithstanding, I think this remedy may frequently be employed with great advantage, as it not only allays irritation, but induces sleep, which the patient is generally deprived of, whilst the stricture continues. It also stops the vomiting, and arrests, or retards, the progress of diseased action; but, however, as soon as the effects of the opium disappear, all the distressing symptoms return with redoubled force. In a case of the first variety, where the symptoms are very severe, and the surgeon from some cause is constrained to defer the operation, opium may be given with the happiest effects; for exclusive of its anodyne properties, it elevates the pulse, cheers the spirits, and diffuses an additional heat over the surface of the body. If, however, the operation of returning the sac unopened be intended, this medicine had better, in general, be omitted; for if performed whilst the patient is under its immediate agency, it will be more difficult to ascertain the removal of the stricture, as is exemplified in Case V.

As

As to the result of the operation of the taxis, by pressure, as formerly practiced, and continued uniformly to the present time, being a course of nearly twenty years, I can say, I have succeeded in half the cases I have met with; but whether this practice, on being compared with that of others, be such as to recommend it to general notice, I cannot say. One advantage it seems to possess, namely, that it is productive of no active injury to the parts implicated in the hernia. One disadvantage, however, it may be exposed to, which is, delay, or loss of time, necessarily incurred by the operation; but this inconvenience may be nearly obviated, by the surgeon prescribing to himself a fixed time, of moderate duration, such as before specified. Another result of this practice is, I have been more successful, *cæteris paribus*, in early life, than in advanced age, in women than in men, and in hernias recently strangulated, oftener than those of longer duration.

The danger formerly attending the operation for the strangulated hernia, rather than the difficulty of performing it, suggested a variety of means for its reduction. A whole routine of these was employed, and often repeated, before the surgeon thought himself warranted in exposing his patient to so great a peril. Hence, that procrastination, which involved in itself such fatal consequences.

Instead of trifling away time, by employing and repeating the means usually recommended, and which afford a very small and uncertain prospect of success, and doubtfully compensating the injury arising from delay, we ought to adopt the few prompt remedies which experience has confirmed to be the most efficacious; and if these fail, the operation should be no longer delayed. For this principle we ought never to lose sight of, namely, in proportion as the disease advances, less chance there will be of the operation succeeding; and the plan generally adopted, previous to the operation, when pursued to its usual extent, is of such a debilitating nature, as cannot fail rendering the operation still more precarious.

The reason why my practice, in the preceding, as well as in the other cases in the sequel, were not in unison with this theory, was, from the particular desire I had of giving full trial to pressure; and from the conviction, at the same time, I had of my patient's sustaining very little injury; but I must confess, in future, I shall be less tempted to expose a patient to any risk from procrastination, as I am persuaded, that our principal dependence on pressure is within the first hour of its application.

It is to be recollected, that this practice of long continued pressure, was first founded on the idea, that the stricture, or more particularly the tendon of the oblique muscle, was capable of dilatation and contraction. If any doubt of this theory at that time remained, it has been subsequently done away, by a circumstance that will be related in the case of Susanna Hamshaw.

CASE. II.—Thomas Marsden, aged 78 years, of an athletic form, and who had enjoyed a good state of health the greatest part of his life, was seized with symptoms of strangulated hernia, the 18th of June 1806. Two days after this event, I first saw him, and found him suffering great distress. The vomiting was frequent, and his belly was painful, and rather tense. He was thirsty, but his tongue was not much furred, and he also had not had a stool since the strangulation commenced. The hernial swelling was of the femoral kind, was large and painful to the touch, and of an elliptical form, whose greatest diameter extended in an horizontal direction, and consequently nearly parallel to Poupart's ligament. He said, the first symptom of strangulation was perceived on his getting over a style, but the hernia he had been troubled with fifty-eight years, though not always in the same degree. His pulse was at 90, soft, and contracted; he moaned much, appeared dejected, and had frequent incitements to make water. Fifteen grains of the vitriolated magnesia, dissolved in a table spoonful of water, were ordered him every half hour; but this did not agree well with his stomach, and was accordingly discontinued. Six drops of the tinct. opii, diluted with a small portion of water, were next given him every ten minutes. This seemed to agree well with him, and evidently relieved his sickness and vomiting. Gentle pressure was had recourse to, and continued for three hours, but without success. The tobacco clyster and the operation were both proposed, but neither of which were acceded to. On taking my leave of this patient, I recommended cold applications to the swelling, together with occasional pressure. I afterwards learnt, he called in another practitioner, and that he languished about ten days, six of which he was troubled with a diarrhœa, which only terminated with his existence.

CASE III.—Robert Green, a healthy man, aged 65, and who had been afflicted with a scrotal hernia about two years, was seized with symptoms of strangulation on the 20th of August 1806. On the 24th I first saw him, and found he had been four days in great distress from retching ing and vomiting, which were accompanied with great pain all over the abdomen. He was costive, his tongue somewhat furred, and rather parched, and was thirsty, with a pulse at 75. The hernia was down in the scrotum, was somewhat large, but bore handling well. A linen cloth wrung out of the aq. litharg. acet. comp. was applied to the tumour, on which gentle pressure was also made by the hand for seventeen minutes, when the hernia retired, with no other indication than a diminution of the tumour. Previously, I had ordered him six drops of the tinct. opii to be taken as in the preceding case. After the first dose his sickness and vomiting considerably abated. On the following day I found this man without any thirst, or pain in the abdomen, yet his pulse had increased both in force and velocity, being now full, and at 96; but something of this kind I had formerly observed, after the reduction of that species of strangulated hernia, comprising the first class of symptoms, as before mentioned.

Case IV.—Susanna Hamshaw, a widow woman, aged 68, and who had been subject to an inguinal hernia for several years, was on the morning of the 10th of January 1808, attacked with symptoms of strangulation. These were a violent pain in the right groin, which extended up to the stomach, and all over the abdomen, and attended with excessive sickness, which was succeeded almost immediately by a stool. In this distress she continued all the rest of the day; but had not vomited till within three hours of my first seeing her, yet in this interval she had thrown up frequently. I visited her fourteen hours after the commencement of the strangulation, and found her complaining of great pain in the before mentioned parts. The swelling in the groin extended down to the labia pudendi, and was painful, as well as the rest of the abdomen, on being handled. Her pulse was tense, and at 104, her thirst considerable, but her tongue was tolerably clean. She made water well; in short, her principal complaints were excessive pain and sickness. I gently pushed up the tumour to the abdominal aperture, and retained it in this position by gentle pressure for four minutes and a half, when it receded with slight borborigmi.

There was no medicine given in this case, nor any other means used, save pressure, and in performing which, I was constrained by the position of the patient, to retain the hernia with two of my fore-fingers and thumb, instead of following the directions heretofore given. These, as soon as the reduction took place, were pushed along with the skin, within the opening, (for she was a very thin subject) and I could plainly perceive the ring to contract almost immediately around them.

Case V.—Sarah Taylor, a married woman, aged 46 years, and of a delicate constitution, and who had been troubled with a femoral hernia twenty-six years, was, on the 13th of October 1809, seized with symptoms of strangulation, which were succeeded by two motions. Five days after this event, I saw her, and found since the first attack of strangulation, she had vomited five or six times every twenty-four hours, and in the intervals had frequent returns of sickness, attended with acrid eructations. The hernia was large, but bore handling well, and her belly was rather tense and not much painful to the touch; her pulse was at 100, and in force; her thirst considerable, and her tongue was furred, and appeared as if stained with ink; her urine also was in small quantities, and rather high coloured. I made pressure on the tumour for an hour, and recommended it to be repeated for half an hour at a time, every four hours, during the night; as from circumstances I was prevented from performing the operation till the following day. The aq. litharg. acet. c. was ordered to be applied frequently in the form of stupes, and six drops of the tinct. opii to be taken every ten minutes, for two hours only, along, occasionally, with a small quantity of the vitriolated magnesia dissolved in water.

19th. Her pulse was at 108; her thirst considerable; her tongue rather more furred, and her belly was somewhat inflated, and rather more tense, but not much painful on pressure. The convulsive and acrid eructations much the same as yesterday, but had not vomited since she took the opium, though appeared often sickly, and there also was little alteration in the appearance of the hernia. Her countenance was however considerably changed, and cold clammy sweats were beginning to be perceptible on the extremities.

In this situation the operation was proposed and submitted to with great reluctance. After denuding the hernial sac, I passed my fore-finger, between it and the aponeurosis, with very little resistance, except when I introduced it within Gimbernat's ligament, which was divided as in Case 1. After this I pressed gently on the sac with my fingers, for about half a dozen seconds, when I perceived something to give way, and on which the patient cried out out as if something hurt her. The hernial swelling now became less by more than one half. A florid suffusion overspread her countenance, and which was soon after succeeded by perspiration. Her eyes became more lively and elevated within their sockets, and though laid upon a table, the greatest part of the time she was asleep, a visitation she had not experienced since the commencement of the strangulation. Although I was satisfied that the stricture was removed, yet the gentleman who took a part with me in the operation, still having some doubts, founded on the circumstance of the convulsive eructations remaining undiminished, notwithstanding no other symptoms of strangulation had appeared for the space of an hour. On the ground of this objection, I thought it right to proceed with opening the sac, which was horizontally elongated thus,  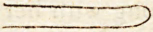 The fundus pointing towards the os ilium, and containing a portion of omentum in a diseased state, more thickened and indurated than natural, and in one place to the size of a small sixpence, dark coloured. About half way between the fundus of the sac and Gimbernat's ligament was a greater quantity of omentum, apparently sound, enveloping a certain portion of intestine, except a space of the magnitude of a small shilling, which was uncovered. This part of the intestine I gently pinched up with my finger and thumb, and with the fingers of my other hand broke the adhesions, which united the intestine and omentum. These were however slight and not extensive. This being accomplished, I pushed up the intestine very gently with my fore finger, and it entered the abdomen, and was followed by the principal part of that portion of omentum which surrounded it; but the remainder at the bottom of the sac adhered so firmly to the anterior part of it, as to induce me to leave it within the wound, which it rather more than completely filled. Dry lint, and a small digestive pledget were the dressings, and these were secured by straps of adhesive plaster, with a view of preventing any protrusion of the hernia; over the whole, also was applied a proper bandage.

20th. Pulse 103; thirst considerable, and her tongue equally furred as yesterday. Her belly was rather more tense and inflated, but she had however slept tolerably in the night. Ordered a table spoonful of the castor oil to be taken every three hours, interposing now and then a little of a solution of the magnes. vitriolat. These medicines procured three loose stools in the course of the day, which which relieved her belly much, but still the belchings were nearly as troublesome and frequent as ever.

21st. Pulse 92, and slept tolerably in the night at intervals. Her tongue was rather better, and her thirst some little abated, but her belly continued yet rather tense and painful. She had three motions this day from her medicines.

22d. Pulse 94, and in other respects much the same. She had had five stools since yesterday, which, together with the eructations, she complained of as being very hot.

23d. Her pulse was at 90, and her belly rather softer, and less sore. Her thirst was some little abated, and her tongue appeared rather less furred. The eructations continued hot and troublesome, and she had only one loose stool in the course of the day. Ordered a little magnesia alba to be taken frequently along with her liquids.

24th. Pulse 90, and in other respects no variation. She had five loose motions to day. The dressings were removed for the first time, and the wound looked well, but the appearance of the omentum was more gangrenous. Dry lint dipped in one part of the ol. terebinth. and two parts of the bals. copaiv. were applied, and a similar pledget and straps as before. In this manner the wound was dressed daily. On the ninth day, the gangrenous portion of omentum sloughed off, and appeared to be about two or three drachms in weight, and to have increased in a ten-fold degree since the operation. The wound was healed in seven weeks. It was sixteen or seventeen days before the pulse subsided to its natural standard, and before her tongue became clean, and her belly soft and flaccid. Her eructations continued some little time longer, and after having apparently left her, were easily re-excited for several months, on any thing greasy being taken into the stomach.

One circumstance attending this case is of some importance, and although already mentioned, yet I will beg leave to recapitulate it. On dividing the stricture, the pulse was at 108, and in an hour afterwards, being at the time of opening the sac, it was reduced to 101, and at the expiration of a second hour it remained stationary, but in the space of other fourteen hours they had advanced to 103. These circumstances tend to confirm so much of the above theory, as relates to the sudden diminution of morbid symptoms, on removal of the stricture, when the sac is unopened, and vice versâ.

I have no doubt that on Gimbernat's ligament being divided, divided, the intestine, and that part of the omentum which surrounded it, were returned, and shortly after descended, on an effort to go to stool; for on opening the sac, the tumour had increased in size, equal to what it was before the stricture was divided.

Another circumstance attending this case, and worth remarking is, the ink-like appearance of the tongue. Could the suppression of the catamenia, for three months previous to the operation, account for this unusual phenomenon?

